# A genetic variation in microRNA target site of *ETS2* is associated with clinical outcomes of paclitaxel-cisplatin chemotherapy in non-small cell lung cancer

**DOI:** 10.18632/oncotarget.7433

**Published:** 2016-02-17

**Authors:** Mi Jeong Hong, Shin Yup Lee, Jin Eun Choi, Cheng Cheng Jin, Hyo Jung Kang, Sun Ah Baek, So Yeon Lee, Kyung Min Shin, Ji Yun Jeong, Won Kee Lee, Seung Soo Yoo, Jaehee Lee, Seung Ick Cha, Chang Ho Kim, Ji Woong Son, Jae Yong Park

**Affiliations:** ^1^ Departments of Biochemistry and Cell Biology, Kyungpook National University Medical Center, Daegu, Republic of Korea; ^2^ Cell and Matrix Research Institute, Kyungpook National University Medical Center, Daegu, Republic of Korea; ^3^ Department of Internal Medicine, School of Medicine, Kyungpook National University, Daegu, Republic of Korea; ^4^ Lung Cancer Center, Kyungpook National University Medical Center, Daegu, Republic of Korea; ^5^ Department of Radiology, Kyungpook National University Medical Center, Daegu, Republic of Korea; ^6^ Department of Pathology, Kyungpook National University Medical Center, Daegu, Republic of Korea; ^7^ Biostatistics Center, School of Medicine, Kyungpook National University, Daegu, Republic of Korea; ^8^ Department of Internal Medicine, Konyang University Hospital, Daejeon, Republic of Korea

**Keywords:** non-small cell lung cancer, miRNA target sites, polymorphisms, chemotherapy, response

## Abstract

The present study was performed to investigate the association of single nucleotide polymorphisms (SNPs) located in the miRNA target sites with the clinical outcomes of first line paclitaxel-cisplatin chemotherapy in advanced NSCLC. Eighty SNPs in miRNA binding sites of cancer related genes selected from 18,500 miRNA:target bindings in crosslinking, ligation, and sequencing of hybrids (CLASH) data were investigated in 379 advanced NSCLC patients using a sequenom mass spectrometry-based genotype assay. qRT-PCR and luciferase assay were conducted to examine functional relevance of potentially functional SNPs in miRNA binding sites. Of the 80 SNPs analyzed, 16 SNPs were significantly associated with the clinical outcomes after chemotherapy. Among these, *ANAPC1* rs3814026C>T, *ETS2* rs461155A>G, *SORBS1* rs7081076C>A and *POLR2A* rs2071504C>T could predict both chemotherapy response and survival. Notably, *ETS2* rs461155A>G was significantly associated with decreased *ETS2* mRNA expression in both tumor and paired normal lung tissues (*Ptrend* = 4 × 10^−7^, and 3 × 10^−4^, respectively). Consistently, a decreased expression of the reporter gene for the G allele of rs461155 compared with the A allele was observed by luciferase assay. These findings suggest that the four SNPs, especially *ETS2* rs461155A>G, could be used as biomarkers predicting the clinical outcomes of NSCLC patients treated with first-line paclitaxel-cisplatin chemotherapy.

## INTRODUCTION

MicroRNAs (miRNAs) are endogenous small (∼22 nucleotides) non-coding RNAs that repress target gene expression by complementary binding to the 3′-untranslated region (3′-UTR) of messenger RNAs (mRNAs), leading to translational repression or mRNA cleavage [1, 2]. The miRNAs play important roles in various biological functions such as cell proliferation and survival, DNA repair, and immune response [3–5]. Evidence indicates that miRNAs are critically involved in the development and progression of diverse human cancers as tumor suppressors and/or oncogenes depending on the tissue type and the presence of specific targets [5–8].

Many studies have suggested that single nucleotide polymorphisms (SNPs) in miRNA target sites are associated with the risk and the prognosis of diverse types of cancer, including lung cancer [9–12]. Most of the studies investigated miRNA binding sites identified by computational prediction methods such as miRanda and TargetScan [13–15]. However, those were developed to predict miRNA-mRNA binding based primarily on the complementarity to seed sequence, being biased towards canonical seed pairings at 3′ UTR, and may have frequent false prediction [13–15]. Recently, crosslinking, ligation, and sequencing of hybrids (CLASH) provided direct experimental observation of transcriptome-wide miRNA-target pairs, revealing that the interactions occurred more frequently in coding sequence than 3′ UTR and the majority of miRNA-target bindings were noncanonical [13].

Based on the important roles of miRNA network in carcinogenesis, we hypothesized that polymorphisms in miRNA target sites may influence miRNA-mRNA binding and consequently the expression of target genes, thereby influencing the response and survival after chemotherapy in lung cancer. To test this hypothesis, we selected SNPs in miRNA binding sites using CLASH data and evaluated their association with the clinical outcome of first line paclitaxel-cisplatin chemotherapy in non-small cell lung cancer (NSCLC).

## RESULTS

### Patient characteristics and clinical predictors

The clinico-pathological characteristics and the association with chemotherapy response and OS are shown in Table 1. The overall response rate was 47.5%. We observed events (deaths) in 347 of the 379 patients (91.6%) and median survival time (MST) was 13.2 months (95% CI = 12.5–14.7 months). The response to chemotherapy was not associated with any of the clinical characteristics. Age, gender, smoking status, tumor histology, weight loss, and second line chemotherapy were significantly associated with the OS (Table 1).

**Table 1:** Univariate analysis for response to chemotherapy and overall survival by clinical variables

Variables	No. of cases	Response to chemotherapy				Overall survival				
		responders^a^ (CR+PR)	nonresponders^a^ (SD+PD)	OR (95% CI)	*P*	MST (months)	95% CI	Log-Rank *P*	HR (95% CI)	*P*
Overall	379	180 (47.5)^a^	199 (52.5)			13.2	12.5-14.7			
Age (years)										
< 65	179	93 (52.0)	86 (48.0)	1.00		15.7	13.7-17.7		1.00	
≥ 65	200	87 (43.5)	113 (56.5)	0.71 (0.48-1.07)	0.10	11.9	10.8-13.2	0.003	1.38(1.11-1.70)	0.003
Gender										
Male	309	153 (49.5)	156 (50.5)	1.00		12.8	11.9-14.3		1.00	
Female	70	27 (38.6)	43 (61.4)	0.64 (0.38-1.09)	0.10	16.8	12.8-22.6	0.02	0.73(0.56-0.96)	0.02
Smoking status										
Never	63	27 (42.9)	36 (57.1)	1.00		19.6	13.7-30.4		1.00	
Ever	316	153 (48.4)	163 (51.6)	1.25 (0.73-2.16)	0.42	12.8	11.7-14.2	0.001	1.59(1.20-2.12)	0.002
Histological type										
Squamous cell ca.	184	98 (53.3)	86 (46.7)	1.00		13.2	11.7-14.4		1.00	
Adenoca.	172	71 (41.3)	101 (58.7)	0.62 (0.41-0.94)	0.02	15.1	12.1-17.5		0.74(0.59-0.92)	0.01
Large cell ca.	23	11 (47.8)	12 (52.2)	0.80 (0.34-1.92)	0.62	11.4	7.4-12.9	0.01	1.25(0.80-1.99)	0.34
Clinical stage										
III	159	82 (51.6)	77 (48.4)	1.00		14.7	12.8-17.4		1.00	
IV	220	98 (44.6)	122 (55.5)	0.75 (0.50-1.14)	0.18	12.7	10.8-14.2	0.12	1.18(0.96-1.47)	0.12
PS ECOG										
0-1	310	149 (48.1)	161 (51.9)	1.00		14.1	12.6-15.7		1.00	
2	69	31 (44.9)	38 (55.1)	0.88 (0.52-1.49)	0.64	12.6	9.7-13.2	0.42	1.12(0.85-1.48)	0.42
Weight loss										
No	233	116 (49.8)	117 (50.2)	1.00		14.4	12.5-16.6		1.00	
Yes	146	64 (43.8)	82 (56.2)	0.79 (0.52-1.19)	0.26	12.9	11.6-14.0	0.001	1.47(1.18-1.83)	0.001
2^nd^ line Chemotherapy										
No	132					11.0	8.1-12.8		1.00	
Yes	247					15.1	13.2-16.6	0.02	0.76(0.61-0.95)	0.02
Radiation to tumor										
No	340					12.9	11.6-14.3		1.00	
Yes	39					18.5	14.0-23.9	0.19	0.80(0.57-1.12)	0.19

Abbreviation: CR, complete response; PR, partial response; SD, stable disease; PD, progressive disease; OR, odds ratio; MST, median survival time; CI, confidence interval; HR, hazard ratio; PS, performance status; ECOG, Eastern Cooperative Oncology Group.

a Row percentage.

### Associations between SNPs and clinical outcomes

**Figure 1:**
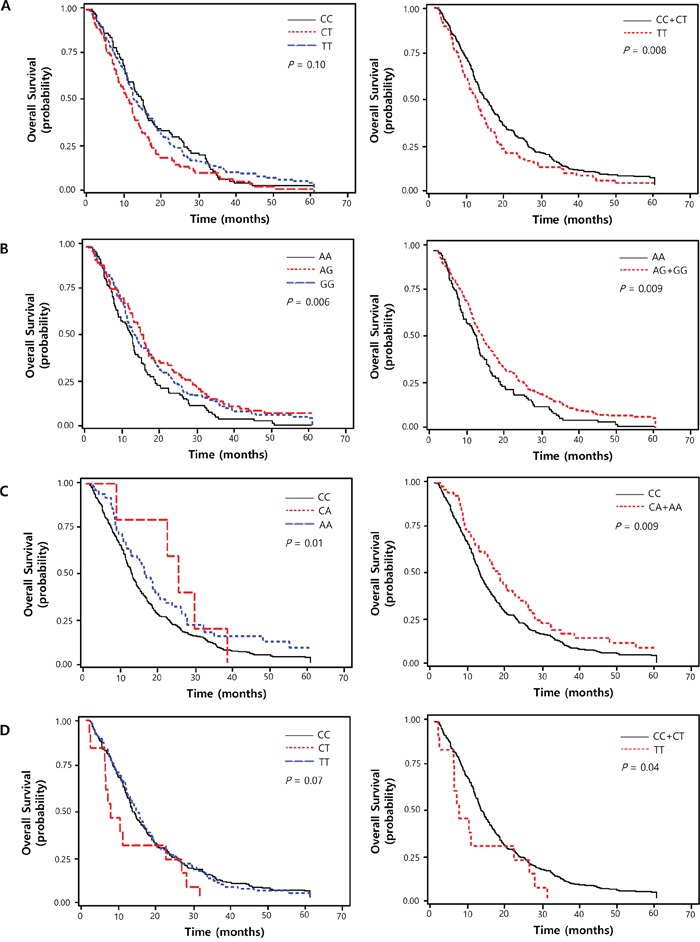
Kaplan-Meier plot of overall survival curves according to (A) ANAPC1 rs3814026C>T, (B) *ETS2* rs461155A>G, (C) *SORBS1* rs7081076C>A and (D) *POLR2A* rs2071504C>T genotypes *P* values in multivariate Cox proportional hazard model.

Among the 100 SNPs genotyped, 80 were analyzed in this study after excluding 5 SNPs with genotype failure and 15 showing deviation from Hardy-Weinberg equilibrium (*P* < 0.05). The SNP ID, gene information, miRNA, and minor allele frequencies are shown in Supplementary Table 1. Of the 80 SNPs analyzed, 16 SNPs listed in Table 2 were significantly associated with chemotherapy response and/or survival. Among these, *ANAPC1* rs3814026C>T, *ETS2* rs461155A>G, *SORBS1* rs7081076C>A and *POLR2A* rs2071504C>T were found to be associated with both chemotherapy response and survival (adjusted OR [aOR] = 0.61, 95% CI = 0.37–1.00, *P* = 0.05; adjusted HR [aHR] = 1.41, 95% CI = 1.10–1.82, *P* = 0.008, under recessive model, respectively; aOR = 1.34, 95% CI = 1.00–1.81, *P* = 0.05; aHR = 0.80, 95% CI = 0.69–0.94, *P* = 0.006, under additive model, respectively; aOR = 2.32, 95% CI = 1.28–4.24, *P* = 0.006; aHR = 0.67, 95% CI = 0.49–0.91, *P* = 0.009, under dominant model; aOR = 0.17, 95% CI = 0.04–0.78, *P* = 0.02; aHR = 1.83, 95% CI = 1.03–3.26, *P* = 0.04, under recessive model, respectively; Table 3 and Figure 1).

**Table 2:** Summary of sixteen SNPs and response to chemotherapy and overall survival

ID No. ^a^	Target Gene	miRNA	Alleles	CR(%)	MAF	HWE-*p*	*P* for response ^b^			*P* for overall survival ^c^		
							dominant	recessive	additive	dominant	recessive	additive
rs3814026	*ANAPC1*	hsa-miR-744	CT	95.26	0.49	0.46	0.48	0.05	0.45	0.91	0.008	0.10
rs461155	*ETS2*	hsa-miR-149	AG	97.63	0.46	0.72	0.05	0.22	0.05	0.009	0.05	0.006
rs7081076	*SORBS1*	hsa-miR-320a	CA	97.63	0.08	0.10	0.006	0.18	0.005	0.009	0.28	0.01
rs2071504	*POLR2A*	hsa-miR-652	CT	95.00	0.19	0.95	0.46	0.02	0.14	0.18	0.04	0.07
rs2228128	*POLR2A*	has-miR-744	TC	98.42	0.07	0.07	0.81	0.97	0.82	0.03	0.38	0.03
rs2261988	*UHRF1*	has-miR-615-3p	CA	98.42	0.15	0.20	0.73	0.99	0.43	0.13	0.01	0.29
rs7091596	*PARD3*	hsa-miR-93*	AT	97.63	0.24	0.55	0.40	0.33	0.29	0.99	0.02	0.33
rs6698826	*RAB3B*	hsa-miR-27b	CA	97.89	0.11	0.48	0.41	0.85	0.41	0.06	0.07	0.04
rs3088440	*CDKN2A*	hsa-miR-10b	GA	98.16	0.09	0.62	0.44	0.99	0.24	0.48	3 × 10^−5^	0.23
rs1140034	*ADCK2*	has-let-7b	TC	98.42	0.07	0.44	0.63	0.99	0.73	0.65	4 × 10^−5^	0.77
rs3217933	*CCND2*	hsa-miR-17	TC	97.89	0.10	0.69	0.04	0.77	0.06	0.17	0.94	0.20
rs2229534	*ACADS*	hsa-miR-92a	GA	96.84	0.20	0.19	0.01	0.40	0.08	0.92	0.61	0.92
rs157705	*MAP3K7*	hsa-miR-1226*	GA	98.68	0.32	0.34	0.03	0.06	0.01	0.59	0.70	0.81
rs1965024	*SALL1*	hsa-miR-423-5p	TC	95.79	0.32	0.46	0.01	0.22	0.01	0.94	0.17	0.51
rs2076345	*TCEB3*	hsa-miR-320b	CT	98.95	0.26	0.63	0.02	0.87	0.04	0.91	0.90	0.97
rs296888	*HNRNPK*	hsa-miR-615-3p	CT	98.42	0.26	0.06	0.03	0.06	0.01	0.27	0.08	0.11
r40311	*GSPT1*	hsa-miR-183	GC	98.42	0.19	0.73	0.04	0.20	0.03	0.27	0.94	0.36

Abbreviation: CR, call rate; MAF, minor allele frequency; and HWE, Hardy-Weinberg equilibrium.

a Information about SNPs and SNP ID were obtained from NCBI database (http://ncbi.nih.gov). The transcription start site was counted as +1 in reference sequences.

b *P* values were calculated by multivariate regression analysis, adjusted for age, gender, smoking status, tumor histology, stage, ECOG performance status, and weight loss.

c *P* values were calculated using multivariate Cox proportional hazard models, adjusted for age, gender, smoking status, tumor histology, stage, ECOG performance status, weight loss, 2nd line chemotherapy and radiation to primary tumor.

**Table 3:** Genotypes of *ANAPC1*, *ETS2*, *SORBS1*, and *POLR2A* polymorphisms and their associations with the response to chemotherapy and overall survival

Polymorphism/Genotype	Target Gene	miRNA	No. of cases (%)^a^	Response				Overall survival			
				Responders (%)^b^	Non-responders (%)^b^	OR (95% CI)^c^	*P*^c^	MST (95% CI)	*L-R P*	HR (95% CI)^d^	*P*^d^
rs3814026^e^	*ANAPC1*	hsa-miR-744									
CC	(UTR-5)		96(26.6)	43(44.8)	53(55.2)	1.00		15.6(11.7-17.1)		1.00	
CT			174(48.2)	93(53.5)	81(46.6)	1.47(0.88-2.46)	0.14	13.7(12.3-16.9)		0.90(0.69-1.18)	0.46
TT			91(25.2)	35(38.5)	56(61.5)	0.79(0.43-1.43)	0.43	12.0 (9.0-13.4)	0.09	1.32(0.97-1.80)	0.08
Dominant						1.19(0.73-1.92)	0.48	12.8(11.9-14.6)	0.52	1.01(0.79-1.31)	0.91
Recessive						0.61(0.37-1.00)	0.05^f^	14.3(12.8-16.4)	0.03	1.41(1.10-1.82)	0.008^j^
Additive						0.89(0.67-1.20)	0.45			1.15(0.98-1.35)	0.10
rs461155^e^	*ETS2*	hsa-miR-149									
AA	(cds-synon)		109(29.5)	43(39.5)	66(60.6)	1.00		12.6 (9.7-13.5)		1.00	
AG			181(48.9)	90(49.7)	91(50.3)	1.51(0.92-2.48)	0.10	13.7(11.7-17.0)		0.77(0.60-0.99)	0.04
GG			80(21.6)	43(53.8)	37(46.3)	1.77(0.98-3.22)	0.06	15.6(12.7-17.7)	0.03	0.65(0.48-0.89)	0.007
Dominant						1.59(1.00-2.53)	0.05	14.3(12.7-16.4)	0.01	0.73(0.58-0.92)	0.009
Recessive						1.37(0.83-2.28)	0.22	12.9(11.7-14.3)	0.13	0.77(0.59-1.01)	0.05
Additive						1.34(1.00-1.81)	0.05^g^			0.80(0.69-0.94)	0.006^k^
rs7081076^e^	*SORBS1*	hsa-miR-320a									
CC	(cds-non)		313(84.6)	140(44.7)	173(55.3)	1.00		12.9(11.9-14.3)		1.00	
CA			52(14.1)	32(61.5)	20(38.5)	2.18(1.17-4.04)	0.01	16.8(11.6-20.4)		0.68(0.49-0.93)	0.02
AA			5(1.4)	4(80.0)	1(20.0)	5.55(0.53-58.4)	0.15	25.4 (8.8-38.4)	0.10	0.57(0.23-1.42)	0.23
Dominant						2.32(1.28-4.24)	0.006^h^	18.1(12.6-22.5)	0.03	0.67(0.49-0.91)	0.009^l^
Recessive						4.99(0.48-51.7)	0.18	13.2(12.5-14.7)	0.39	0.61(0.24-1.51)	0.28
Additive						2.22(1.28-3.85)	0.005			0.70(0.53-0.92)	0.01
rs2071504^e^	*POLR2A*	hsa-miR-652									
CC	(cds-synon)		237(65.8)	116(49.0)	121(51.1)	1.00		13.2(12.3-15.1)		1.00	
CT			110(30.6)	53(48.2)	57(51.8)	0.98(0.62-1.57)	0.94	14.3(11.8-16.8)		1.12(0.87-1.45)	0.38
TT			13(3.6)	2(15.4)	11(84.6)	0.16(0.04-0.78)	0.02	7.4 (5.8-22.1)	0.23	1.89(1.06-3.38)	0.03
Dominant						0.84(0.54-1.32)	0.46	13.7(11.6-16.4)	0.65	1.18(0.93-1.51)	0.18
Recessive						0.17(0.04-0.78)	0.02^i^	13.7(12.6-15.1)	0.08	1.83(1.03-3.26)	0.04^m^
Additive						0.74(0.50-1.10)	0.14			1.22(0.99-1.50)	0.07

Abbreviation: OR, odds ratio; CI, confidence interval; MST, median survival time (months); *L-R P*, Log-rank *P*; HR, hazard ratio.

a Column percentage.

b Row percentage.

c OR, 95% CI, and their corresponding *P* values were calculated by multivariate regression analysis, adjusted for age, gender, smoking status, tumor histology, stage, ECOG performance status, and weight loss.

d HRs, 95% CIs and their corresponding *P* values were calculated using multivariate Cox proportional hazard model, adjusted for age, gender, smoking status, tumor histology, stage, ECOG performance status, weight loss, 2nd line chemotherapy, and radiation to primary tumor.

e Genotype failure: 18 cases for rs3814026, 9 for rs461155, 9 for rs7081076, 19 for rs2071504.

^f-m^ *P* values by FDR methods:

f 0.25,

g 0.10,

h 0.01,

i 0.06,

j 0.04,

k 0.01,

l 0.03,

m 0.10.

### Effect of SNPs in microRNA target sites on mRNA expression

**Figure 2:**
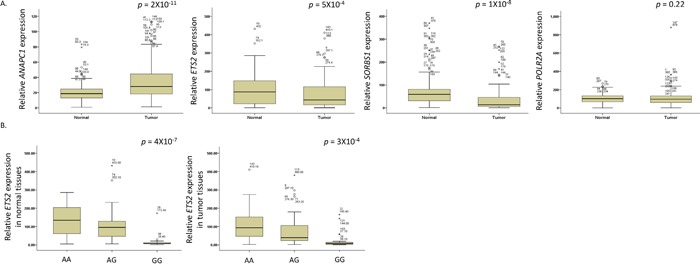
(A) The mRNA expression levels of ANAPC1, ETS2, SORBS1, and POLR2A genes and (B) *ETS2* mRNA expression by the rs461155A>G genotypes, in tumor and non-malignant lung tissues The *ETS2* mRNA expression levels and the association with the rs461155A>G genotypes were examined in tumor (30 AA, 62 AG, and 23 GG) and paired non-malignant lung (37 AA, 67 AG, and 21 GG) tissues. The mRNA expression levels of each gene were normalized with that of β-actin gene. The horizontal lines within the boxes represent the median values; the upper and lower boundaries of the boxes represent 75th and 25th percentiles, respectively; the upper and lower bars indicate the largest and smallest observed values, respectively, except outliers. *P* values, Student's t-test.

To identify the functional effect of *ANAPC1* rs3814026C>T, *ETS2* rs461155A>G, *SORBS1* rs7081076C>A and *POLR2A* rs2071504C>T, we evaluated the relationship between the genotypes of those SNPs and mRNA expression of each gene in tumor and paired non-malignant lung tissues. As shown in Figure 2A, *ANAPC1* expression level was significantly higher (*P* = 2 × 10^−11^), and *ETS2* and *SORBS1* expression level was significantly lower in tumor tissues than in non-malignant tissues (*P* = 5 × 10^−4^ and 1 × 10^−8^), respectively. However, *POLR2A* expression level was not different between tumor and normal tissues. Notably, *ETS2* rs461155A>G was significantly associated with decreased *ETS2* mRNA expression in both tumor and paired normal tissues (*Ptrend* = 4 × 10^−7^, and 3 × 10^−4^, respectively, Figure 2B). The difference among genotypes was not observed in *ANAPC1*, *SORBS1* and *POLR2A* expression (data not shown).

### Effect of SNPs in miRNA target sites on miRNA binding

**Figure 3:**
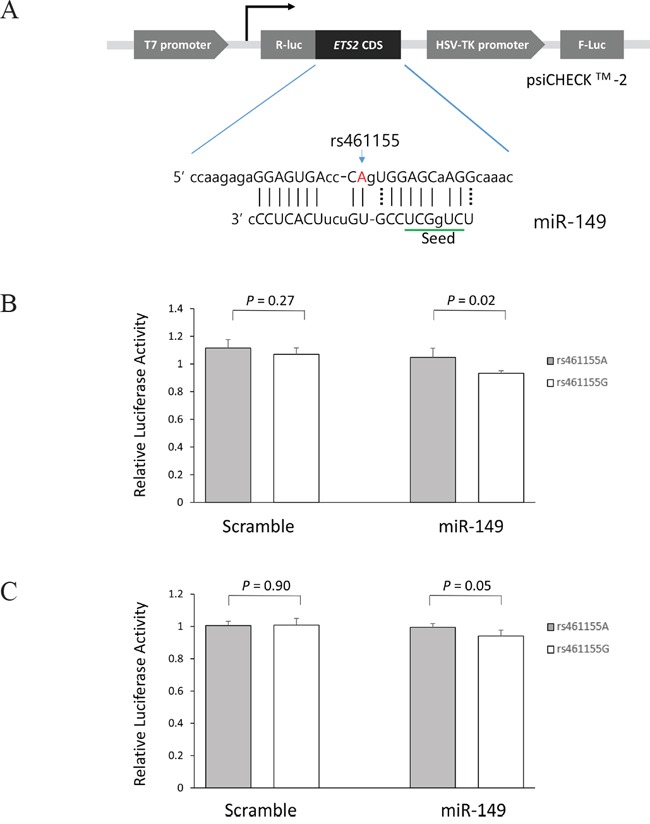
Functional analysis of the ETS2 rs461155A>G by dual luciferase reporter assay **(A)** Schematic representation of reporter plasmids containing *ETS2* rs461155A>G, and the complementarity between miR-149 and *ETS2* coding sequence targeted. *Renilla* luciferase assay for miRNA effect on rs461155A>G polymorphism using **(B)** and H1299 cells **(C)** Cells were co-transfected with hsa-miR-149-5p, and psiCHECK2 plasmid containing *ETS2* 296 bp coding sequence fragments with A or G allele. *Renilla* luciferase activity was normalized to firefly luciferase activity and data are presented relative to the Mock control. Each bar represents mean ± SE from two independent experiments carried out in quadruplicate. *P*-value, Student's t-test. R-luc, *Renilla* luciferase; F-luc, firefly luciferase; HSV-TK, Herpes simplex virus thymidine kinase.

To investigate whether *ETS2* rs461155A>G, a synonymous SNP at coding sequence, modulates the binding of miR-149, and thereby alter the expression of *ETS2* gene, we generated psiCHECK™-2-*ETS2* constructs containing rs461155A>G and co-transfected the constructs into A549 and H1299 cells with miR-149. CLASH data showed that the binding between *ETS2* and miR-149 was noncanonical. The rs461155 caused an A-to-G change at the position which pairs with nucleotide 11 of miR-149, outside the seed region (Figure 3A). As shown in Figure 3B and C, the *Renilla* luciferase activity was significantly decreased in *ETS2* rs461155G compared with *ETS2* rs461155A in both A549 and H1299 cells (*P* = 0.02 and 0.05, respectively), which was consistent with the mRNA expression. These results suggest that rs461155A>G may lead to decreased *ETS2* expression by altering the binding of miR-149 to *ETS2* mRNA.

## DISCUSSION

We investigated the associations between SNPs in miRNA target sites and the treatment outcomes of 1^st^ line paclitaxel-cisplatin chemotherapy, to identify genetic variations that affect the clinical outcomes in NSCLC. Using the CLASH data that provides experimentally proved transcriptome-wide miRNA-target pairs, 80 potentially functional SNPs in miRNA binding sites of cancer-related genes were tested for this study. Four SNPs, *ANAPC1* rs3814026C>T, *ETS2* rs461155A>G, *SORBS1* rs7081076C>A, and *POLR2A* rs2071504C>T, could predict both chemotherapy response and survival. Notably, functional evidence for *ETS2* rs461155A>G was provided by *ETS2* mRNA expression in clinical samples, which was further supported by *in vitro* assays using lung cancer cell lines. These findings suggest that the SNPs, especially *ETS2* rs461155A>G, could be used as predictive/prognostic markers for advanced stage NSCLC receiving paclitaxel-cisplatin chemotherapy. This is the first study investigating the effect of miRNA binding site polymorphisms on chemotherapy outcome in lung cancer.

In the present study, four SNPs in miRNA binding sites were associated with chemotherapy response and survival in patients with advanced stage NSCLC. Among those, the *ETS2* rs461155A>G was strongly correlated with decreased *ETS2* mRNA expression level in both tumor and normal lung tissues. In addition, *in vitro* luciferase assay suggested that the *ETS2* rs461155 A-to-G change could alter binding efficiency of miR-149 to *ETS2* mRNA, leading to decreased *ETS2* mRNA expression level and better clinical outcomes of lung cancer. However, the biologic mechanism of the observed association between the SNP and the clinical outcomes remains unclear because the role of ETS2 in lung cancer has not been fully elucidated. ETS2 is a transcription factor belonging to the ETS family proteins which control gene expression by binding to over 200 target genes with GGAA/T ETS binding site. ETS2 controls the expression of genes that are critical for a wide range of biological processes including cellular proliferation, differentiation, transformation, angiogenesis, apoptosis, and Ras signal transduction [16–18], and is also required for telomerase activity [19]. It has been reported that ETS2 functions as both oncogene [18, 20–23] and tumor-suppressor gene [24–27] in different types of malignancies. Accumulating evidence suggests that the same ETS factor may function either as a positive or as a negative regulator of target gene transcription [16, 28]. Based on the target DNA sequence, binding of an ETS protein near other transcription factors or additional tissue-specific factors and the consequent interaction between them may result in either synergistic activation or repression of specific target genes, suggesting promoter and cell context specificity [29]. Recently, Kabbout et al. reported that ETS2 exerts a tumor suppressor function in NSCLC pathogenesis through inhibition of the MET proto-oncogene, and that low ETS2 expression by immunohistochemistry was a significant predictor of shorter time to recurrence after curative resection in early stage NSCLC [27]. In the present study, however, *ETS2* rs461155 variant G allele, which correlated to decreased expression level of *ETS2* mRNA compared with A allele, was associated with better chemotherapy response and survival of the patients with advanced NSCLC. Given the different settings of the two studies, one possible explanation is that the discordant observation could be attributable to the effect of ETS2 on its target genes involved in DNA repair or cell cycle regulation, because alteration of those genes may lead to discordant outcomes in terms of cancer progression and chemotherapy response [30–33]. For example, DNA repair genes have been linked to protection against development and progression of cancer, thereby better prognosis, whereas they have also been associated with resistance to platinum-based anticancer drugs and poor prognosis [30–31]. Further investigation is needed to understand the role of ETS2 in lung cancer and the mechanism of its dissimilar effects on the prognosis of patients in different stages undergoing different anticancer therapy.

Using CLASH data for the association study, this study provides a unique opportunity to evaluate SNPs in miRNA targets outside 3′ UTR and in noncanonical miRNA-mRNA binding in terms of functional and clinical consequences in NSCLC. Although the four SNPs were associated with chemotherapy response and survival, considering the borderline CIs of some associations and multiple comparisons issue with a modest sample size, the impact of the SNPs on chemotherapy outcomes would be marginal. However, our study provides functional evidence for the association of *ETS2* rs461155A>G with clinical outcomes by showing its strong correlation with *ETS2* mRNA expression in clinical samples as well as *in vitro* assays using lung cancer cell lines, which supports the credibility of the association. Further studies are warranted to validate our results in a larger population with diverse ethnicity.

In conclusion, this study suggests that the four SNPs, especially ETS2 rs461155A>G, could be used as biomarkers predicting the clinical outcomes of NSCLC patients treated with first-line paclitaxel-cisplatin chemotherapy. Future studies are needed to confirm our findings and to understand the biologic function of *ETS2* in the development and progression of lung cancer.

## MATERIALS AND METHODS

### Study population and chemotherapy

The study population has been described in our previous study [34]. In brief, 379 patients with stage III or IV NSCLC treated with at least two cycles of paclitaxel-cisplatin chemotherapy as a first-line treatment at Kyungpook National University Hospital (KNUH) in Daegu, Korea between August 2005 and December 2008. Genomic DNA samples from the patients were provided by the National Biobank of Korea, KNUH, which is supported by the Ministry of Health, Welfare and Family Affairs. Patients who underwent concurrent chemoradiotherapy were excluded to avoid the confounding effect of radiation on chemotherapy response. The chemotherapy regimen consisted of paclitaxel 175 mg/m^2^ administered i.v. over three hours, and cisplatin 60 mg/m^2^ infused over one hours on day 1, every three weeks. Treatment was discontinued upon disease progression, major toxicities, or according to patient decision or physician discretion. Assessment of tumor response was performed by computed tomography scan every two cycles. Responses were assessed using Response Evaluation Criteria in Solid Tumors [35]. The best overall response for each patient was reported and all responses were reviewed by an independent radiologist. Patients with a complete response (CR) or a partial response (PR) were defined as responders, and patients having stable disease (SD) or progressive disease (PD) were defined as nonresponders. For the assessment of survival outcomes, overall survival (OS), defined as the time between the date of chemotherapy start and the date of death or last follow-up, were recorded. There were 184 squamous cell carcinomas (SCC), 172 adenocarcinomas (AC), 3 large cell carcinomas, and 20 cases classified as unspecified NSCLC. Fifty nine patients had stage IIIA disease, 100 stage IIIB, and 220 stage IV. Written informed consent was obtained from all patients and this study was approved by the Institutional Review Board of the KNUH.

### SNP selection and genotyping

We searched for all the potentially functional polymorphisms in miRNA target sites using PolymiRTS database 3.0 (http://compbio.uthsc.edu/miRSNP; ref. 36), and selected 24,027 SNPs in experimentally validated miRNA target sites by downloading data from CLASH experiment which has been integrated in PolymiRTS database 3.0 (last modified in July 2013). Among those, 1,574 SNPs in cancer-related genes were selected using a downloaded list of cancer genes from the CancerGenes database (http://cbio.mskcc.org/cancergenes; ref. 37). Finally 100 SNPs with the minor allele frequency (MAF) ≥ 0.05 in the HapMap JPT data were collected after excluding those in linkage disequilibrium (LD, *r* ^2^ ≥ 0.8). Genotyping was performed using Sequenom MassARRAY® iPLEX assay (Sequenom Inc., San Diego, CA). For validation of genotyping, approximately 5% of samples were randomly selected and genotyped again with a restriction fragment length polymorphism assay by a different investigator and the results were 100% concordant.

### RNA preparation and quantitative reverse transcription-PCR

*Anaphase promoting complex subunit 1 (ANAPC1), V-ets avian erythroblastosis virus E26 oncogene homolog 2 (ETS2), Sorbin and SH3 domain containing 1 (SORBS1), and Polymerase (RNA) II (DNA directed) polypeptide A, 220kDa (POLR2A)* mRNA expression was measured by quantitative reverse transcription-PCR. Total RNA was isolated from paired tumor and nonmalignant lung tissues of 154 NSCLC patients who underwent surgery in Kyungpook National University Medical Center between September 2011 and August 2014 using TRIzol (Invitrogen, Carlsbad, CA) and reverse transcribed using the QuantiTect reverse transcription kit (QIAGEN, Hilden, Germany). Real time-PCR was performed for each gene and beta-actin with QuantiFast SYBR Green PCR Master Mix (QIAGEN) in a LightCycler 480 (Roche Applied Science, Mannheim, Germany) using the following primers: ANAPC1 forward, 5′-CGCGTCCCGAGTTATACAG-3′; ANAPC1 reverse, 5′-TCTCGACCAAAAGGAACAAATTC-3′; ETS2 forward, 5′-CAGATGTTCCCCAAGTCTCG-3′; ETS2 reverse, 5′-GTCGTGGTCTTTGGGAGTC-3′; SORBS1 forward, 5′-CAGTTCAGAGCCCCAGTTG-3′; SORBS1 reverse, 5′-CTTTGTCTTGCCCATTGCTG-3′; POLR2A forward, 5′-CAAGTATGGCATGGAGATCCC-3′; POLR2A reverse, 5′-TGGTTCCAAGGTGTCATGG-3′; beta-actin forward, 5′-TTGTTACAGGAAGTCCCT TGCC-3′; beta-actin reverse, 5′-ATGCTATCACC TCCCCTGTGT-3′. Each test was carried out according to standard protocol. Relative each gene expression was calculated following normalization with human beta actin. The relative mRNA expression were normalized with β-actin expression and then calculated by the 2^−ΔΔCT^ method [38].

### Cloning of the luciferase reports gene and dual luciferase assay

We investigated whether miR-149, which was reportedly bind to *ETS2* mRNA in CLASH experiment [13] and known to be expressed in lung cancer [39, 40], changes the *ETS2* expression level on its binding sites including rs461155A>G by luciferase report assay. The psiCHECK™-2 vector (Promega, Madison, WI, USA) was used to construct luciferase reporter plasmids. Briefly, the putative target site *ETS2* coding sequence containing rs461155A or rs461155G was synthesized by PCR from human genomic DNA and cloned into the psiCHECK™-2 vector. The construct was confirmed by DNA sequencing. The NSCLC cell lines, H1299 and A549, were purchased from Korean Cell Line Bank (KCLB), Seoul, Korea, and authenticated by KCLB using short tandem repeat DNA fingerprinting. The cells were plated in 24-well plates at a density of 5×10^4^ cells/well. The following day, cells were transfected with construct vector and 30 nM miScript miRNA Mimic (Syn-hsa-miR-149-5p; QIAGEN, Hilden, Germany) or AllStars negative control siRNA (QIAGEN, Hilden, Germany) based on the manufacturer's instructions. After 24 h of incubation, Firefly and *Renilla* luciferase activities were measured using the Dual-Luciferase® Reporter Assay System (Promega, Madison, WI, USA). The *Renilla* luciferase values were normalized using firefly luciferase activity and data are presented relative to the Mock control. All experiments were performed twice in quadruplicate.

### Statistical analysis

Hardy-Weinberg equilibrium was tested using a goodness-of-fit χ^2^ test with 1 *degree of freedom*. The genotypes for each SNP were analyzed as a three-group categorical variable, and those were also grouped according to the dominant and recessive model. The association between clinical variables or genotypes and chemotherapy response was tested by odds ratio (OR) and 95% confidence intervals (CIs) using unconditional logistic regression analysis. The survival estimates were calculated using the Kaplan-Meier method. The difference in OS according to different clinical variables or genotypes was compared using log-rank tests. Cox's proportional hazard regression model was used for the multivariate survival analyses. The hazard ratio (HR) and 95% confidence interval (CI) were also estimated. A cut-off *p*-value of 0.05 was adopted for all the statistical analyses. The statistical data were obtained using Statistical Analysis System for Windows, version 9.2 (SAS Institute, Cary, NC, USA).

## SUPPLEMENTARY TABLE




