# Supplementary material for: A genetic variation in microRNA target site of ETS2 is associated with clinical outcomes of paclitaxel-cisplatin chemotherapy in non-small cell lung cancer
Source: Oncotarget. 2016 Feb 17;7(13):15948–58. doi: 10.18632/oncotarget.7433 (PMC4941289; doi:10.18632/oncotarget.7433)
Supplement: Supplementary file 1 [file oncotarget-07-15948-s001.pdf]

SUPPLEMENTARY TABLE

Supplementary Table 1: Eighty SNPs in miRNA target sites identified by CLASH and evaluated for chemotherapy response and survival

See Supplementary File 1
