# Supplementary material for: A genetic variation in microRNA target site of ETS2 is associated with clinical outcomes of paclitaxel-cisplatin chemotherapy in non-small cell lung cancer
Source: Oncotarget. 2016 Feb 17;7(13):15948–58. doi: 10.18632/oncotarget.7433 (PMC4941289; doi:10.18632/oncotarget.7433)
Supplement: Supplementary file 2 [file oncotarget-07-15948-s002.docx]

| Supplementary Table 1. Eighty SNPs in miRNA target sites identified by CLASH and evaluated for chemotherapy response and survival | | | | | | | | | | |
| --- | --- | --- | --- | --- | --- | --- | --- | --- | --- | --- |
|  |  |  |  | *P* for response ^b^ | | |  | *P* for overall survival ^c^ | | |
| ID No. ^a^ | Target Gene | miRNA | MAF^b^ | Dominant | Recessive | Additive |  | Dominant | Recessive | Additive |
| rs3814026C>T | *ANAPC1* | hsa-miR-744 | 0.49 | 0.48 | 0.05 | 0.45 |  | 0.91 | 0.01 | 0.10 |
| rs461155A>G | *ETS2* | hsa-miR-149 | 0.46 | 0.05 | 0.22 | 0.05 |  | 0.01 | 0.05 | 0.01 |
| rs7081076C>A | *SORBS1* | hsa-miR-320a | 0.08 | 0.01 | 0.18 | 0.00 |  | 0.01 | 0.28 | 0.01 |
| rs2071504C>T | *POLR2A* | hsa-miR-652 | 0.19 | 0.46 | 0.02 | 0.14 |  | 0.18 | 0.04 | 0.07 |
| rs2228128T>C | *POLR2A* | has-miR-744 | 0.07 | 0.81 | 0.97 | 0.82 |  | 0.03 | 0.38 | 0.03 |
| rs2261988C>A | *UHRF1* | has-miR-615-3p | 0.15 | 0.73 | 0.99 | 0.43 |  | 0.13 | 0.01 | 0.29 |
| rs7091596A>T | *PARD3* | hsa-miR-93* | 0.24 | 0.40 | 0.33 | 0.29 |  | 0.99 | 0.02 | 0.33 |
| rs1049434A>T | *SLC16A1* | hsa-miR-615-3p | 0.33 | 0.58 | 0.10 | 0.23 |  | 0.06 | 0.06 | 0.47 |
| rs6698826C>A | *RAB3B* | hsa-miR-27b | 0.11 | 0.41 | 0.85 | 0.41 |  | 0.06 | 0.07 | 0.03 |
| rs3088440G>A | *CDKN2A* | hsa-miR-10b | 0.09 | 0.44 | 0.99 | 0.24 |  | 0.48 | <.0001 | 0.23 |
| rs1140034T>C | *ADCK2* | has-let-7b | 0.07 | 0.63 | 0.99 | 0.73 |  | 0.65 | <.0001 | 0.77 |
| rs3217933T>C | *CCND2* | hsa-miR-17 | 0.10 | 0.04 | 0.77 | 0.06 |  | 0.17 | 0.94 | 0.2 |
| rs2229534G>A | *ACADS* | hsa-miR-92a | 0.20 | 0.01 | 0.40 | 0.08 |  | 0.92 | 0.61 | 0.92 |
| rs157705G>A | *MAP3K7* | hsa-miR-1226* | 0.32 | 0.03 | 0.06 | 0.01 |  | 0.59 | 0.7 | 0.81 |
| rs1965024T>C | *SALL1* | hsa-miR-423-5p | 0.32 | 0.01 | 0.22 | 0.01 |  | 0.94 | 0.17 | 0.51 |
| rs2076345C>T | *TCEB3* | hsa-miR-320b | 0.26 | 0.02 | 0.87 | 0.04 |  | 0.91 | 0.9 | 0.97 |
| rs296888C>T | *HNRNPK* | hsa-miR-615-3p | 0.26 | 0.03 | 0.06 | 0.01 |  | 0.27 | 0.08 | 0.11 |
| rs40311G>C | *GSPT1* | hsa-miR-183 | 0.19 | 0.04 | 0.20 | 0.03 |  | 0.27 | 0.94 | 0.36 |
| rs3182911T>C | *COPS3* | hsa-miR-484 | 0.36 | 0.55 | 0.49 | 0.94 |  | 0.2 | 0.92 | 0.4 |
| rs9479G>A | *PML* | hsa-miR-378 | 0.36 | 0.85 | 0.11 | 0.35 |  | 0.65 | 0.21 | 0.34 |
| rs2230724G>A | *JAK2* | hsa-miR-16 | 0.38 | 0.36 | 0.27 | 0.91 |  | 0.19 | 0.37 | 0.16 |
| rs14235A>G | *BCKDK* | hsa-miR-92b | 0.09 | 0.42 | 0.99 | 0.61 |  | 0.28 | 0.05 | 0.4 |
| rs2295865C>A | *SUPT16H* | hsa-miR-186 | 0.10 | 0.32 | 0.87 | 0.33 |  | 0.81 | 0.74 | 0.88 |
| rs1061157G>A | *BMPR2* | hsa-miR-93* | 0.08 | 0.25 | 0.32 | 0.40 |  | 0.7 | 0.93 | 0.7 |
| rs7654C>A | *TPM3* | hsa-miR-615-3p | 0.07 | 0.25 | 0.86 | 0.34 |  | 0.4 | 0.45 | 0.61 |
| rs1653586G>T | *CAMKK2* | hsa-miR-185 | 0.07 | 0.13 | 0.91 | 0.17 |  | 0.79 | 0.98 | 0.8 |
| rs3826810G>A | *LDLR* | hsa-miR-92a | 0.10 | 0.11 | 0.55 | 0.21 |  | 0.89 | 0.61 | 1.0 |
| rs4919551C>G | *BTRC* | hsa-let-7e | 0.11 | 0.72 | 0.98 | 0.73 |  | 0.64 | 0.74 | 0.61 |
| rs1136613G>A | *KPNB1* | hsa-miR-484 | 0.12 | 0.73 | 0.59 | 0.65 |  | 0.96 | 0.53 | 0.83 |
| rs20554G>A | *EP300* | hsa-miR-23b | 0.15 | 0.38 | 0.34 | 0.62 |  | 0.42 | 0.57 | 0.61 |
| rs4074826C>T | *HIPK2* | hsa-miR-423-5p | 0.17 | 0.07 | 0.72 | 0.10 |  | 0.85 | 0.05 | 0.35 |
| rs2288539C>T | *NR2F6* | has-miR-196a | 0.18 | 0.83 | 0.52 | 0.96 |  | 0.92 | 0.73 | 0.84 |
| rs1056471G>C | *HADHB* | hsa-miR-99a | 0.17 | 0.26 | 0.72 | 0.27 |  | 0.81 | 0.28 | 0.87 |
| rs7195830G>A | *CYBA* | hsa-miR-320a | 0.20 | 0.92 | 0.63 | 0.78 |  | 0.53 | 0.93 | 0.59 |
| rs3203733A>G | *MTUS1* | hsa-miR-1296 | 0.24 | 0.87 | 0.41 | 0.64 |  | 0.09 | 0.33 | 0.09 |
| rs1047840G>A | *EXO1* | hsa-miR-30e | 0.23 | 0.68 | 0.69 | 0.62 |  | 0.59 | 0.88 | 0.69 |
| rs1707303G>T | *PIK3R3* | hsa-miR-346 | 0.26 | 0.82 | 0.20 | 0.74 |  | 0.46 | 0.55 | 0.39 |
| rs16855615G>A | *CCNT2* | hsa-miR-484 | 0.26 | 0.76 | 0.99 | 0.80 |  | 0.93 | 0.59 | 0.88 |
| rs5744857A>G | *POLE* | hsa-miR-92a | 0.30 | 0.71 | 0.91 | 0.74 |  | 0.4 | 0.95 | 0.5 |
| rs6116042A>T | *CDC25B* | hsa-miR-296-3p | 0.34 | 0.86 | 0.98 | 0.89 |  | 0.27 | 0.78 | 0.5 |
| rs2230738A>G | *ADCY9* | hsa-miR-877* | 0.35 | 0.12 | 0.79 | 0.21 |  | 0.88 | 0.56 | 0.69 |
| rs2074216G>A | *DVL2* | has-miR-484 | 0.37 | 0.44 | 0.59 | 0.78 |  | 0.87 | 0.06 | 0.31 |
| rs8444C>T | *LASS2* | hsa-miR-20a | 0.39 | 0.95 | 0.99 | 0.97 |  | 0.64 | 0.3 | 0.38 |
| rs12449580C>G | *AIPL1* | has-miR-3615 | 0.38 | 0.87 | 0.27 | 0.50 |  | 0.92 | 0.26 | 0.53 |
| rs1051709T>C | *PTK7* | hsa-miR-193b | 0.42 | 0.69 | 0.96 | 0.81 |  | 0.77 | 0.31 | 0.72 |
| rs2854464A>G | *ACVR1B* | hsa-miR-182 | 0.43 | 0.26 | 0.23 | 0.16 |  | 0.31 | 0.35 | 0.23 |
| rs1480153T>C | *PPP2R2B* | hsa-miR-30e* | 0.48 | 0.23 | 0.22 | 0.15 |  | 0.64 | 0.28 | 0.35 |
| rs7097A>G | *POLR1D* | hsa-miR-374a* | 0.48 | 0.68 | 0.56 | 0.92 |  | 0.39 | 0.45 | 0.32 |
| rs3212986G>T | *CD3EAP* | hsa-miR-92a | 0.25 | 0.79 | 0.38 | 0.56 |  | 0.53 | 0.96 | 0.6 |
| rs4705C>T | *PDGFRL* | hsa-miR-25 | 0.48 | 0.81 | 0.58 | 0.86 |  | 0.76 | 0.24 | 0.37 |
| rs362331T>C | *HTT* | has-let-7b | 0.47 | 0.30 | 0.22 | 0.17 |  | 0.28 | 0.43 | 0.24 |
| rs2306409T>C | *GTPBP4* | hsa-miR-16 | 0.42 | 0.69 | 0.67 | 0.62 |  | 0.85 | 0.92 | 0.86 |
| rs1058028T>C | *ETV6* | hsa-miR-99b | 0.43 | 0.79 | 0.11 | 0.48 |  | 0.41 | 0.55 | 0.83 |
| rs9523A>G | *NUP62* | hsa-miR-1914 | 0.42 | 0.97 | 0.19 | 0.46 |  | 0.77 | 0.54 | 0.89 |
| rs2269529C>T | *MYH9* | hsa-miR-1296 | 0.41 | 0.27 | 0.95 | 0.44 |  | 0.58 | 0.5 | 0.46 |
| rs6934058T>C | *CDC5L* | hsa-miR-505 | 0.44 | 0.60 | 0.10 | 0.56 |  | 0.75 | 0.13 | 0.28 |
| rs989902A>C | *PTPN13* | hsa-miR-186 | 0.36 | 0.27 | 0.84 | 0.36 |  | 0.86 | 0.42 | 0.61 |
| rs2277559A>G | *BUB1B* | hsa-miR-193b | 0.33 | 0.94 | 0.07 | 0.36 |  | 0.91 | 0.14 | 0.57 |
| rs2589668C>T | *42069* | hsa-let-7b | 0.32 | 0.12 | 0.28 | 0.50 |  | 0.96 | 0.81 | 0.88 |
| rs2297441G>A | *RTEL1* | hsa-miR-615-3p | 0.29 | 0.95 | 0.89 | 0.91 |  | 0.34 | 0.64 | 0.6 |
| rs1111667A>G | *ERO1LB* | hsa-miR-106b* | 0.27 | 0.79 | 0.97 | 0.82 |  | 0.62 | 0.6 | 0.54 |
| rs6089219T>G | *CABLES2* | has-miR-106b* | 0.29 | 0.87 | 0.64 | 0.74 |  | 0.96 | 0.26 | 0.58 |
| rs1713982G>A | *POLR2B* | hsa-miR-615-3p | 0.25 | 0.76 | 0.46 | 0.94 |  | 0.88 | 0.73 | 0.79 |
| rs1049910C>G | *LMNB2* | hsa-miR-193b | 0.29 | 0.28 | 0.09 | 0.11 |  | 0.29 | 0.44 | 0.59 |
| rs2279130C>T | *ARRB1* | hsa-miR-92a | 0.25 | 0.49 | 0.90 | 0.55 |  | 0.8 | 0.78 | 0.92 |
| rs1013062C>T | *DEK* | hsa-miR-96 | 0.27 | 0.24 | 0.22 | 0.15 |  | 0.1 | 0.89 | 0.21 |
| rs6757T>C | *BSG* | hsa-miR-92a | 0.23 | 0.26 | 0.54 | 0.50 |  | 0.3 | 0.73 | 0.33 |
| rs11196251C>T | *TCF7L2* | hsa-miR-324-5p | 0.20 | 0.35 | 0.34 | 0.26 |  | 0.13 | 0.07 | 0.06 |
| rs1318648T>G | *ESPL1* | hsa-miR-149 | 0.24 | 0.62 | 0.54 | 0.85 |  | 0.82 | 0.21 | 0.54 |
| rs2290890G>A | *SYK* | hsa-miR-99b | 0.18 | 0.72 | 0.29 | 0.93 |  | 0.12 | 0.94 | 0.2 |
| rs12620338G>A | *PAX3* | hsa-miR-324-5p | 0.22 | 0.28 | 0.24 | 0.18 |  | 0.98 | 0.41 | 0.77 |
| rs1136666C>G | *GAPDH* | has-miR-149 | 0.19 | 0.30 | 0.08 | 0.14 |  | 0.71 | 0.98 | 0.76 |
| rs7116130A>G | *PSMD13* | hsa-miR-93* | 0.19 | 0.31 | 0.76 | 0.34 |  | 0.21 | 0.76 | 0.25 |
| rs1569238G>A | *REPS1* | hsa-miR-193b | 0.20 | 0.22 | 0.33 | 0.17 |  | 0.08 | 0.31 | 0.07 |
| rs1046025G>A | *PSMD6* | hsa-miR-331-3p | 0.19 | 0.99 | 0.96 | 0.98 |  | 0.87 | 0.81 | 0.95 |
| rs3786362T>C | *TYMS* | hsa-miR-615-3p | 0.14 | 0.82 | 0.39 | 0.65 |  | 0.73 | 0.66 | 0.84 |
| rs3741328G>A | *DDX6* | hsa-miR-877* | 0.09 | 0.58 | 0.60 | 0.52 |  | 0.23 | 0.85 | 0.27 |
| rs7488A>G | *TPP1* | hsa-miR-877 | 0.12 | 0.40 | 0.45 | 0.33 |  | 0.67 | 0.86 | 0.72 |
| rs4623769G>A | *PCTK3* | hsa-miR-222 | 0.06 | 0.54 | 0.98 | 0.71 |  | 0.95 | 0.24 | 0.86 |
| rs3762158G>C | *SUPT16H* | has-miR-484 | 0.10 | 0.47 | 0.88 | 0.47 |  | 0.55 | 0.69 | 0.64 |

Abbreviation: MAF, minor allele frequency.

^a^ Information about SNPs and SNP ID were obtained from NCBI database ([http://ncbi.nih.gov](http://ncbi.nih.gov/)). The transcription start site was counted as +1 in reference sequences.

^b^ *P* values were calculated by multivariate regression analysis, adjusted for age, gender, smoking status, tumor histology, stage, ECOG performance status, and weight loss.

^c^ *P*-values were calculated using multivariate Cox proportional hazard models, adjusted for age, gender, smoking status, tumor histology, stage, ECOG performance

status, weight loss, 2nd line chemotherapy and radiation to primary tumor.
